# Implementation and dissemination of a transition of care program for rural veterans: a controlled before and after study

**DOI:** 10.1186/s13012-017-0653-1

**Published:** 2017-10-23

**Authors:** Chelsea Leonard, Emily Lawrence, Marina McCreight, Brandi Lippmann, Lynette Kelley, Ashlea Mayberry, Amy Ladebue, Heather Gilmartin, Murray J. Côté, Jacqueline Jones, Borsika A. Rabin, P. Michael Ho, Robert Burke

**Affiliations:** 1 Denver/Seattle Center of Innovation for Veteran-Centered and Value Driven Care, VA Eastern Colorado Healthcare System, 1055 Clermont Street, Denver, 80220 CO USA; 2 Department of Health Policy and Management, School of Public Health, Texas A&M University, College Station, 77843 TX USA; 3 College of Nursing, University of Colorado Anschutz Medical Campus, 13001 E 17th Pl, Aurora, 80045 CO USA; 4 Department of Family Medicine and Public Health, University of California San Diego, La Jolla, 92093 CA USA; 5 Division of Cardiology, Department of Medicine, School of Medicine, University of Colorado Denver, 13001 E 17th Pl, Aurora, 80045 CO USA; 6 Hospital Medicine Section, Denver VA Medical Center, 1055 Clermont St, Denver, 80220 CO USA

**Keywords:** Transitions of care, Veterans, Rural health, Implementation, Adaptation, Dissemination, PRISM

## Abstract

**Background:**

Adapting promising health care interventions to local settings is a critical component in the dissemination and implementation process. The Veterans Health Administration (VHA) rural transitions nurse program (TNP) is a nurse-led, Veteran-centered intervention designed to improve transitional care for rural Veterans funded by VA national offices for dissemination to other VA sites serving a predominantly rural Veteran population. Here, we describe our novel approach to the implementation and evaluation = the TNP.

**Methods:**

This is a controlled before and after study that assesses both implementation and intervention outcomes. During *pre-implementation*, we assessed site context using a mixed method approach with data from diverse sources including facility-level quantitative data, key informant and Veteran interviews, observations of the discharge process, and a group brainstorming activity. We used the Practical Robust Implementation and Sustainability Model (PRISM) to inform our inquiries, to integrate data from all sources, and to identify factors that may affect implementation. In the *implementation phase*, we will use internal and external facilitation, paired with audit and feedback, to encourage appropriate contextual adaptations. We will use a modified Stirman framework to document adaptations. During the *evaluation phase*, we will measure intervention and implementation outcomes at each site using the RE-AIM framework (Reach, Effectiveness, Adoption, Implementation, and Maintenance). We will conduct a difference-in-differences analysis with propensity-matched Veterans and VA facilities as a control. Our primary intervention outcome is 30-day readmission and Emergency Department visit rates. We will use our findings to develop an implementation toolkit that will inform the larger scale-up of the TNP across the VA.

**Discussion:**

The use of PRISM to inform pre-implementation evaluation and synthesize data from multiple sources, coupled with internal and external facilitation, is a novel approach to engaging sites in adapting interventions while promoting fidelity to the intervention. Our application of PRISM to pre-implementation and midline evaluation, as well as documentation of adaptations, provides an opportunity to identify and address contextual factors that may impede or enhance implementation and sustainability of health interventions and inform dissemination.

## Background

Patients are at high risk for adverse events during the transition period from hospital to home [1, 2]. The transition home presents challenges such as adjusting to new or changed medications, understanding disease management strategies, and learning home care routines. “Bridging” (i.e., pre- and post-discharge) interventions to improve transitional care practices reduce hospital readmissions [3]. These interventions focus on creating an “ideal” transition of care, which includes preparing patients for discharge with thorough education about self-care, ensuring the completion of medication reconciliation, arranging for any home care needs, and organizing appropriate follow-up [3–9]. Improving the transition from hospital to home is especially important in high-risk cohorts [3, 10], such as the elderly or those who live in a rural setting.

Rural Veterans are a high-risk cohort as they face barriers in nearly all aspects of an ideal transition in care. One such barrier relates to the structure of the Veterans Health Administration (VA) healthcare system. The VA is organized in a hub and spoke system, with large hospitals in urban areas and affiliated smaller clinics in rural areas. This structure leads to unique challenges for rural Veterans transitioning back to their primary care provider, such as difficulties with medication reconciliation between VA facilities, medication gaps due to limited availability of specialized medications in rural areas, inadequate discharge plans, lack of communication between tertiary hospitals and rural Patient Aligned Care Team (PACT) sites, and missed follow-up appointments with primary care providers (PCPs) [11]. In this paper, we describe the dissemination and implementation plan for the rural Transitions Nurse Program (TNP), a nurse-led and Veteran-centered intervention designed to address these transitional care gaps and improve post-discharge outcomes for rural Veterans hospitalized at tertiary VA sites.

The dual goals of this project are to (1) improve transitions and reduce hospital readmissions for rural Veterans hospitalized at tertiary VA facilities, and (2) contribute to the field of implementation science through a robust application of the PRISM framework at every stage from pre-implementation contextual assessment, to implementation and adaptation, to evaluation. The PRISM framework has been used to assess site context in a number of studies [12, 13], but to our knowledge, it has not been used to organize and integrate data from multiple sources or in an iterative manner to guide implementation and evaluation in successive waves. This work will address a gap in the literature on the use of PRISM to guide implementation. It will test the utility of PRISM to guide scale-up efforts of an intervention to diverse settings and provide an example of an innovative diffusion process in the VA.

## Methods

### Description of intervention

The TNP is an intervention carried out with a Transition Nurse (TN) in collaboration with a hospitalist site champion at each intervention site. The intervention is based on the Ideal Transitions of Care (ITC) framework [3] and consists of four core components. These are (1) pre-discharge assessment of patient understanding of self-care, medications, supports for post-discharge care, and obtaining a follow-up appointment; (2) structured post-discharge interactive communication between the hospital and primary care team alerting the primary care team of discharge; (3) a follow-up call to the patient within 48 to 72 h of discharge to confirm attendance to the follow-up appointment with the PCP, reinforce medication and self-care education provided at discharge, and assess symptoms and concerns; and (4) build knowledge and capacity at both the tertiary and primary care site to deliver improved care to future rural Veterans. The TNP was piloted in Denver between 2014 and 2016 with encouraging results such as reduced hospital readmissions and reduced costs relative to a control population [11]. Based on the success of the pilot, the TNP was funded by the VA Office of Rural Health with the support of the Veterans Health Administration (VHA) Office of Nursing Services for expansion to additional tertiary VA sites over the next 5 years.

### Intervention and study design

**Fig. 1:**
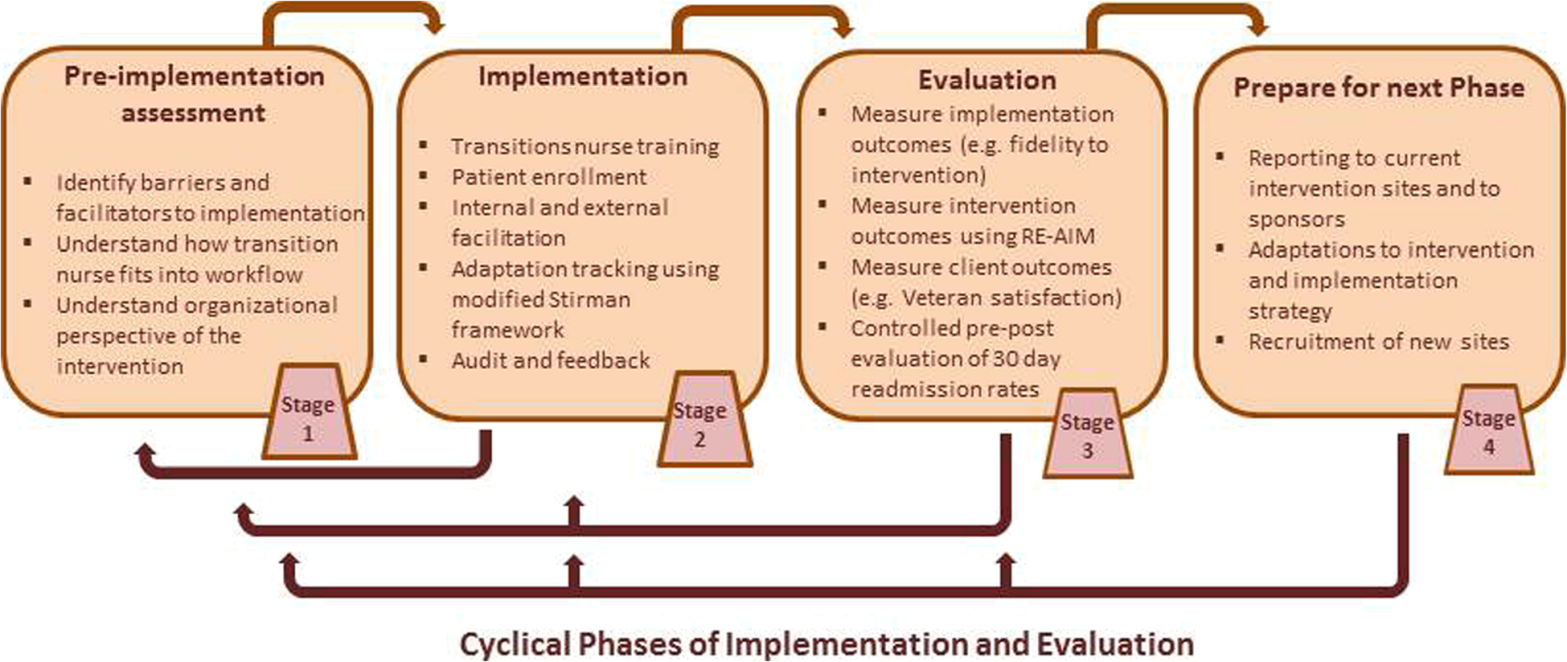
TNP flow chart. This figure illustrates how each stage of the TNP intervention informs subsequent stages. Our pre-implementation evaluation of intervention recipients informs adaptations to the intervention. Evaluation of adaptations and intervention outcomes inform preparation for the next cycle of implementation

We will use the Practical, Robust, Implementation, and Sustainability (PRISM) model [14] to inform the both implementation and evaluation of TNP. PRISM is ideal for guiding our multi-site implementation effort because it accounts for multi-level effects; it builds on several Implementation Science frameworks and can guide pre-implementation, implementation, and evaluation. Several studies show that the adoption, implementation, and sustainment of interventions are related to how well the intervention is integrated into the local context [12–16]. We will assess PRISM domains using convergent mixed methods, collecting both quantitative facility-level data, and qualitative data. We will use PRISM in each phase of our intervention: pre-implementation, implementation, and evaluation. Figure 1 shows the flow of project phases.

This is a type II hybrid study, which tests both intervention outcomes and the implementation strategy [17]. TNP implementation will occur in waves, enrolling additional sites every 12 months. Each wave of implementation will follow three stages: pre-implementation training and evaluation, implementation, and outcome evaluation. It is important to distinguish the evaluation components of pre-implementation and implementation from the final outcome evaluation. Pre-implementation evaluation will be used to evaluate the implementation strategy and tailor the intervention to each site, formative midline evaluation 6 months after implementation will measure the initial intervention outcomes and provide performance feedback to each site, whereas summative outcome evaluation will measure the overall success of the program outcomes and implementation strategy at each site, and will be used to adapt future waves of implementation.

### Context

In the first wave of implementation, the program will be implemented at five sites (2017), one site from each of five geographic VA “regions,” the North Atlantic Region, the Southeast Region, the Midwest Region, the Continental Region, and the Pacific Region. These are tertiary hospital sites that treat more than 1000 rural Veterans annually, had leadership willing to sign a letter of support, and an active hospitalist program that cares for the majority of medical inpatients at the tertiary site. Prior to the second wave of implementation, we will evaluate implementation at the first five sites to test the utility of our implementation strategy. In subsequent waves, the Office of Rural Health will use a national solicitation of applicants for participation in the TNP through a formal application process. Sites that serve large rural populations will be encouraged to apply through targeted promotional materials to hospital leadership.

### Description of implementation strategy

We will conduct pre-implementation site assessments before rolling out the program at each expansion site. Our implementation strategy combines robust pre-implementation work with internal and external facilitation, and audit and feedback.

#### Pre-implementation

During the pre-implementation phase, we will collect qualitative contextual data to (1) understand the current process for transitioning rural Veterans back to their PCP after an inpatient hospitalization at a VHA tertiary medical center, (2) identify and highlight the impact of factors encapsulated in the PRISM framework that may enhance or discourage the implementation of TNP at participating sites, and (3) use these data to facilitate implementation, document adaptations to the program, and enhance sustainability of the intervention and its implementation. In addition, we will meet with site leadership and identify a hospitalist champion as an internal facilitator. A hospitalist was chosen for this role to provide a link between inpatient and outpatient settings and to help troubleshoot clinical problems with enrolled patients. Each site will identify a cohort of rural patients to enroll in the program and modulate the TN workflow to avoid overlap with other transition of care practices.

Qualitative methods include key informant interviews, process mapping, as well as site visits with adapted ethnographic observation and a written group brainstorming activity (brainwriting). Key informant interviews and process mapping interviews with providers and administrators will begin prior to pre-implementation site visits. Table 1 summarizes how we use these methods to measure PRISM domains in our pre-implementation evaluation.

**Table 1:** Assessing PRISM domains to understand context for implementation

PRISM domain	What we are assessing	Data collection technique
Organizational perspective	▪ Current transition process ▪ How TNP fits in the broader organization ▪ Contextual factors that may impede or enhance TNP implementation	▪ Process mapping interviews ▪ Key informant interviews ▪ Adapted ethnography ▪ Brainwriting activity ▪ VA all employee survey, PACT survey, Pi2 index, inpatient data (IPEC) ▪ Implementation readiness survey
Patient perspective	▪ Current transition process ▪ Satisfaction with transition process ▪ Receptiveness to TN role	▪ Veteran interviews ▪ Adapted mini ethnography
External environment	▪ VA regulations ▪ Existing VA infrastructure (CPRS) ▪ Political climate and funding	▪ Key informant interviews ▪ Brainwriting activity
Implementation and sustainability infrastructure	▪ Existing processes and systems ▪ Current transition process ▪ Existing relationships and collaboration ▪ Plan for sustainability	▪ National level VA quantitative data ▪ Key informant interviews
Organizational characteristics	▪ Management support ▪ Shared goals and cooperation ▪ Inter-facility communication	▪ National level VA quantitative data ▪ Process mapping ▪ Brainwriting ▪ Key informant interviews
Patient characteristics	▪ Demographics ▪ Rural veteran readmission dates	▪ Key informant ▪ Brainwriting ▪ Veteran interviews ▪ Quantitative data on 30-day readmissions

We will employ process mapping to understand the current discharge process for rural Veterans and inform the implementation process [18]. We will validate process maps using adapted ethnographic observations on-site visits [19]. During adapted ethnographic observation, qualitative analysts will join medical teams on rounds and observe the patient discharge process. Team members will collect information on the process, interactions among providers and between providers and patients, and contextual information on the atmosphere. Process maps will also aid in return on the investment (ROI) analyses by identifying each role involved in the discharge process, the steps in the process, and time and resources required to complete each step in the discharge process.

Key informant interviews with providers at VA tertiary hospitals and rural PACT clinics will identify attitudes and beliefs, initial reactions to the TNP, barriers and facilitators to implementation of the TNP, and participant perspectives on the potential value of the program. Interviews will be summarized for each site and used to inform site visit data collection. In particular, interviews will determine which areas of the discharge process need clarification or confirmation and identify remaining questions.

Finally, during pre-implementation site visits, we will conduct a brainwriting activity [20, 21]. The brainwriting activity will be used to clarify the rural patient discharge process at each site, to identify perceived failure points in the implementation of TNP and to consider strategies to overcome these barriers. Site visits provide the opportunity to verify what we learn in key informant and process map interviews.

Pre-implementation data will inform adaptations to the intervention. We will provide sites with initial feedback from our interviews and observations within 1 week of the site visit using rapid qualitative analysis techniques [22, 23]. This will allow sites to begin planning for implementation. Additionally, cross-site barriers and facilitators will be identified and discussed with site transition nurses and hospital champions during pre-implementation training.

### Transition nurse training

Transition nurses from each site will be trained in their role prior to implementation. Transition nurses will complete care coordination and transitions management (CCTM) online training and a 2-day in-person training session in Denver. CCTM is conducted through a course offered by the American Academy of Ambulatory Care Nursing (AAACN) and was created from a consensus statement of the AAACN about the core competencies for care coordination [24]. The in-person training will take place at the Center for Advancing Professional Excellence (CAPE) at the University Of Colorado School Of Medicine in Denver, CO. This 2-day training will focus on learning and practicing relationship-centered communication skills and goal-based effective feedback techniques, through utilization of standardized patients. The skills-based training is interactive, experiential, and grounded in feedback. Training will discuss skills for initiating and maintaining relationships, as well as sharing information and negotiating a mutual plan of action. Pre- and post-training surveys will assess nurse satisfaction and perceived change in confidence and knowledge after training.

#### Implementation

Our implementation strategy uses internal and external facilitation [25] and audit and feedback [25, 26] to encourage real-time adaptation of the intervention to each site. In our intervention, external facilitation will be conducted by a team in Denver. The primary investigator in Denver (RB) will meet with individual site leadership and leverage relationships with the Office of Rural Health and the Office of Nursing Services. Internal facilitation is conducted by the hospitalist champion, who will receive data from the team in Denver and have an on-the-ground role in establishing successful implementation of the program. Each site must complete the four core components of the intervention but will individualize the intervention by determining how the intervention best fits their needs and local context. For instance, each site will be responsible for identifying the high-risk rural patient population they will enroll, as each site has more rural Veteran hospitalizations (more than 1000 annually at each site) than the transition nurse can likely enroll (pilot data suggests enrollment of 250–350 rural Veterans annually).

In the first month of implementation, the TN at each site will participate in a regular teleconference with facilitators in Denver to identify barriers to intervention uptake and formulate solutions. The teleconference will serve to support the TNs as they begin enrolling patients in the program, to continually identify barriers to the program and to promote a community of practice. The Denver team will conduct a site visit 6 months after the initial implementation on TNP. The purpose of this site visit is to understand local context in order to better assist with remaining implementation barriers and to evaluate implementation fidelity. This is part of our audit and feedback strategy. By monitoring implementation at each site and assessing barriers as they arise, we will collect actionable data to share with the hospitalist champions that can be used to customize the TNP to each site. For example, we will share with each site the number of Veterans admitted, the number of Veterans who receive all components of the intervention, the time required to enroll Veterans in the program, and hospital readmission rates on a monthly to quarterly basis.

In addition, the facilitators in Denver will also provide contextual interventions to address problems that may arise during implementation (e.g., problems identified in teleconferences and site visits). Possible interventions for barriers include targeted capacity building, team building, encouraging and facilitating communication, reaching out to leadership to enlist support for the intervention, and providing knowledge sharing opportunities (e.g., best practices).

We will ask the first five sites to facilitate implementation at the second wave of sites using a “train the trainer” approach. We will create a manual that encapsulates lessons learned, common barriers to implementation, and solutions to these barriers and ask the first five sites to consider using this manual to “train” the new sites in their region.

### Implementation outcomes

We will assess both intervention outcomes and implementation outcomes [27, 28]. We will use the RE-AIM framework [29] to measure implementation outcomes (see Table 2).

**Table 2:** RE-AIM measures in the rural transition nurse program

RE-AIM measures	TNP definition	Measurement
R—Reach	▪ Proportion of eligible rural Veterans enrolled at each site	▪ Number of Veterans enrolled each month ▪ Proportion of eligible Vets enrolled
E—Effectiveness	▪ Primary outcome is emergency department visits and hospitalizations in the 30 days following index discharge; Cost of utilization (ED/hospital); Satisfaction of Veterans and providers	▪ Hospital readmission rates ▪ Return on Investment ▪ Provider satisfaction surveys ▪ Qualitative semi-structured interviews ▪ Veteran satisfaction surveys
A—Adoption	▪ Proportion of inpatient providers that refer eligible Veterans to the TN for enrollment in the TNP ▪ Proportion of PACT providers that complete communication (*close communication loop through Lync, email, phone*) for care coordination with the TN as part of the TNP	▪ Number of Veterans enrolled each month ▪ Proportion of eligible Vets enrolled, 6 months after enrollment begins, and 1 year after enrollment begins
I—Implementation	▪ Evaluating what components of the manual and toolkit have been implemented and how they have been adapted (using Stirman framework)	▪ Regular phone calls with TNs will identify adaptations, barriers, and facilitators to implementation ▪ Survey to measure TN and Hospital Champion view of program ▪ Observational assessment of TN competency and adherence to TNP core components, as well and pre and post-test ▪ Semi-structured interviews with TNs, Hospital Champions, inpatient and PACT clinicians, and Veterans to identify internal and external factors that affect TNP implementation, as well as barriers and facilitators to implementation ▪ Measurement of adaptations using real-time tracking with and adapted Stirman framework
M—Maintenance	▪ Funding or expansion of TN role at expansion sites after 3 years of funding	▪ Return on investment ▪ Continued use and improvement of TNP program

We will complement RE-AIM with the use of an adapted version of the Stirman adaptation and modification framework [30] to systematically document program modifications during implementation. Our pragmatic approach emphasizes the need for careful balance between fidelity to core program elements and modifications of peripheral program components (i.e., those components that are not essential to achieving consistent program outcomes) to local circumstances and preferences. The adapted Stirman framework includes tracking who makes modifications, what is modified, at what level of delivery, context modifications, and the nature of context modifications. Using this information in combination with intervention outcome measures, we will determine which parts of the intervention are necessary for success, in order to inform subsequent waves of implementation and to ensure sustainability in present and future contexts.

All qualitative data will be managed and coded using *Atlas.ti* [31] according to PRISM domains. A priori codes were developed through group discussion; additional codes that emerge will be applied to all previously coded manuscripts until saturation is reached [32–34]. Intercoder reliability will be conducted through team-based consensus building; qualitative analysts will independently code the same three transcripts and then discuss points of divergence and convergence. These discussions and coding of additional transcripts will continue until the group reaches consensus on the code meaning and application. Investigators with expertise in qualitative methods and implementation science (JJ, BR) will moderate these discussions.

### Intervention outcomes

Intervention outcomes will gauge the effects of the intervention Veteran health outcomes (e.g., reduction in 30-day readmissions and Emergency Department visits). Due to restrictions of the funding agency, we cannot use a randomized design. Therefore, we are using a rigorous non-randomized design. We will conduct a difference-in-differences analysis using similar VA facilities as a control. Facilities will be matched based on their propensity for intervention assignment. Propensity scores will take into account potential confounders including both summarized patient and facility-level factors such as percent rurality, size, readmission rates, and patient risk factors. Our primary outcome will be 30-day readmission and Emergency Department visit rate. We will compare 30-day readmission and Emergency Department visit rates during a defined pre-intervention period at both intervention and control sites, and again beginning 6 months after implementation. We will also conduct an ROI analysis of the TNP, comparing the costs of implementing the program and the costs of the TN with reductions in post-discharge utilization costs. Finally, we will measure client outcomes like Veteran satisfaction.

## Discussion

The TNP is an innovative intervention with the dual goals of (1) improving the transition of care for rural Veterans hospitalized at tertiary VA hospitals and (2) testing a novel application of PRISM to assess contextual elements and tailor the intervention to individual sites. This study represents a robust application of the PRISM framework to a novel intervention. As a type II hybrid intervention-implementation study, we are also assessing novel implementation strategies combining internal and external facilitation and audit and feedback. Our implementation strategy is unique in its use of PRISM to integrate data from a variety of sources in a pre-implementation evaluation with the goal of providing real-time feedback to sites to enhance the impact adoption, implementation, and impact of the TNP.

The TNP builds on components of existing successful Transition Nurse Programs, such as the Department of Veterans Affairs Coordinated Transitional Care (C-Trac) program [35, 36]. C-Trac utilizes nurse case managers to work with patients on care coordination after discharge to community settings from a VA hospital. Like the TNP, C-Trac involves direct communication between the nurse case manager and patients via a post-discharge follow-up call. TNP builds on this program by promoting relationship building between the TN at VA tertiary hospitals and nursing staff at rural PACT sites. The creators of C-Trac used a modified version of the Replicating Effective Programs (REP) implementation theory model to adapt C-Trac to different contexts. REP is most commonly used at the population level rather than the hospital level and begins with the identification of a local site champion and documentation of existing process, followed by a pre-implementation phase that focuses on stakeholder engagement and coaching, identification of barriers, staff training, and also guiding stakeholders in formally adapting programs and documenting modifications [37]. We feel that PRISM is more appropriate for our intervention because it has a greater focus on understanding local contextual factors and organization, allowing for adaptations to diverse and unique environments. PRISM also includes the RE-AIM framework for evaluation. In our approach, external facilitators monitor the intervention using RE-AIM measures and feed the data to internal facilitators, allowing them to make targeted modifications to the intervention when necessary.

PRISM has proven a useful framework for several interventions. Liles et al. [13] used PRISM to explore internal and external barriers to colorectal cancer screening. They were able to identify barriers to colorectal screening at the staff, provider, and patient levels through stakeholder interviews, and were able to implement a program that addressed these specific barriers. Similarly, Beck et al. [12] used PRISM to assess barriers and facilitators in focus groups. As far as we know, our study is the first application of PRISM to assess context, barriers, and facilitators using data from such a wide variety of sources. Our use of PRISM to integrate data from multiple sources is also unique. We are unaware of another intervention using PRISM as a data reduction tool.

This study represents a robust application of the PRISM framework to a novel intervention. Results of our analysis will inform the utility of the PRISM framework for developing better implementation and evaluation strategies. Our multi-source data will allow us to assess the utility of our pre-implementation strategies and determine which are most successful, and to assess the utility of our intervention training and implementation strategies. Our use of the Stirman framework [34] for tracking modifications to the intervention as well as RE-AIM [33] for measuring program outcomes will allow us to assess the reach, effectiveness, adoption, implementation, and maintenance of the program in real time. In each subsequent wave of implementation, we will be able to use this information to enhance external facilitation and may be able to utilize novel study designs or new strategies when appropriate. Our use of standardized frameworks will make our findings applicable to the development, adaptation, and dissemination of new health interventions.

The primary focus of the TNP is to improve the transition of care for rural Veterans. Successful implementation will lead to reduced hospital readmissions through improved follow-up care and communication between hospitals and rural PACT sites. Our findings will have implications for the development of programs addressing transitions of care for vulnerable patient populations.

## Abbreviations

AAACN: American Academy of Ambulatory Care Nursing
CAPE: Center for Advancing Professional Excellence
CCTM: Care coordination and transitions management
C-Trac: Department of Veterans Affairs Coordinated Transitional Care Program
ITC: Ideal Transitions of Care
PACT: Patient Aligned Care Team
PCP: Primary care provider
PRISM: Practical Robust Implementation and Sustainability Model
RE-AIM: Reach, Effectiveness, Adoption, Implementation, and Maintenance Framework
REP: Replicating Effective Programs
TN: Transitions nurse
TNP: Transitions nurse program
VA: Veterans Health Administration
VHA: Veterans Health Administration
